# The Effect of CYP2D6 Phenotypes on the Pharmacokinetics of Propafenone: A Systematic Review and Meta-Analysis

**DOI:** 10.3390/pharmaceutics14071446

**Published:** 2022-07-11

**Authors:** Quyen Thi Tran, In-hwan Baek, Na-young Han, Hwi-yeol Yun, Jung-woo Chae

**Affiliations:** 1 College of Pharmacy, Chungnam National University, Daejeon 34134, Korea; quyentr911@gmail.com; 2 College of Pharmacy, Kyungsung University, Busan 48434, Korea; baek@ks.ac.kr; 3 College of Pharmacy, Jeju National University, Jeju 63243, Korea; hanny@jejunu.ac.kr; 4 Bio-AI Convergence Research Center, Chungnam National University, Daejeon 34134, Korea

**Keywords:** propafenone, pharmacokinetics, phenotype, CYP2D6, precision medicine

## Abstract

Propafenone (PPF) is a class 1C antiarrhythmic agent mainly metabolized by cytochrome (CYP) 2D6, CYP1A2, and CYP3A4. Previous studies have shown that CYP2D6 polymorphism influences the pharmacokinetics (PK) of PPF. However, the small sample sizes of PK studies can lead to less precise estimates of the PK parameters. Thus, this meta-analysis was performed to merge all current PK studies of PPF to determine the effects of the CYP2D6 phenotype more accurately on the PPF PK profile. We searched electronic databases for published studies to investigate the association between the PPF PK and CYP2D6 phenotype. Four PK-related outcomes were included: area under the time–concentration curve (AUC), maximum concentration (C_max_), apparent clearance (CL/F), and half-life (t_1/2_). A total of five studies were included in this meta-analysis (*n* = 56). Analyses were performed to compare PK parameters between poor metabolizers (PMs) versus extensive metabolizers (EMs). PPF has a non-linear pharmacokinetics; therefore, analyses were performed according to dose (300 mg and 400 mg). At 300 mg, the AUC mean (95% CI), C_max_, and t_1/2_ of PPF in PMs were 15.9 (12.5–19.2) µg·h/mL, 1.10 (0.796–1.40) µg/mL, and 12.8 (11.3–14.3) h, respectively; these values were 2.4-, 11.2-, and 4.7-fold higher than those in the EM group, respectively. At 400 mg, a comparison was performed between S- and R-enantiomers. The CL/F was approximately 1.4-fold higher for the R-form compared with the S-form, which was a significant difference. This study demonstrated that CYP2D6 metabolizer status could significantly affect the PPF PK profile. Adjusting the dose of PPF according to CYP2D6 phenotype would help to avoid adverse effects and ensure treatment efficacy.

## 1. Introduction

Cytochrome P450 (CYP) 2D6 is one of member of the CYP450 gene superfamily. CYP2D6 metabolizes approximately 20–25% of the currently available drugs [1]. CYP2D6 shows a high degree of interindividual variability, resulting in differences among the responses of individuals, including the lack of response to treatment and/or adverse effects at a given dose [1]. CYP2D6 polymorphism is thought to be the cause of these different responses. Many studies have investigated the effects of CYP2D6 polymorphism on the pharmacokinetics (PK) of CYP2D6 substrates [2,3,4,5]. Shu-feng Zhou [5] examined the clinical significance of CYP2D6 polymorphism and found that hepatotoxicity and peripheral neuropathy occurred more frequently in poor metabolizers (PMs) who used perhexiline. The authors also found that healthy PMs treated with carvedilol for 1 week had significantly lower systolic blood pressure. These variations in responses indicate that adjusting the dose of CYP2D6 substrates could be beneficial [2,3,4,6]. Kirchheiner et al. [2] investigated the relation between CYP2D6/CYP2D19 and antidepressants and found that 14 antidepressants had substantial variances in PK parameters, and the authors recommended changing the dose. A 50% dose reduction of tricyclic antidepressants was suggested for PMs.

Propafenone (PPF) is a class 1C antiarrhythmic agent used to prolong the time to recurrence of symptomatic atrial fibrillation in patients with episodic atrial fibrillation who do not have structural heart disease. It is also used to prolong the time to recurrence of paroxysmal supraventricular tachycardia associated with disabling symptoms in patients who do not have structural heart disease and to treat documented life-threatening ventricular arrhythmias [7]. PPF is extensively metabolized by phase I and phase II reactions [8], with its major metabolites being 5-hydroxypropafenone (via CYP2D6) and nor-propafenone (via CYP1A2 and CYP3A4) [9]. Studies have shown that CYP2D6 polymorphism affects the PPF PK profile [10,11,12]. Zhang et al. (2019) classified people with CYP2D6 polymorphism into four groups: ultra-rapid metabolizers (UMs), extensive metabolizers (EMs), intermediate metabolizers (IMs), and poor metabolizers (PMs) [13]. Cai et al. classified people into two main groups, PMs and EMs, where EMs included IMs and very extensive metabolizers [14]. Another way to define the CYP2D6 phenotype is to translate it from the genotype. Subjects carrying two non-functional alleles (i.e., *3, *4, *5, *6) were classified as PMs; those harboring one non-functional allele (*3, *4, *5, *6, *8, etc.) and one decreased-function allele (*9, *10, *17, etc.) were classified as IMs; those who had one functional allele (*1, *2, *33, *35) were classified as EMs; and those carrying two functional alleles were classified as UMs [13,15].

The PK profile of CYP2D6 substrates can be considerably altered on the basis of the CYP2D6 polymorphism. For example, PPF peak concentrations in PMs (737 ± 318 ng/mL) were found to be significantly higher than those in EMs (238 ± 183 ng/mL) [10]. Dilger et al. found that the bioavailability of PPF was approximately eightfold higher in PMs than in EMs (54.3 ± 13.43 nmol·h/mL vs. 6.86 ± 4.39 nmol·h/mL) [16]. The Dutch Pharmacogenetics Working Group of Royal Dutch Pharmacists Association suggested reducing the initial dose of PPF by 70% in CYP2D6 PMs, but there is insufficient data to allow for calculating dose adjustments for the other metabolizer groups [3].

Pharmacokinetic studies often have small sample sizes, resulting in less precise parameter estimates. Therefore, we conducted a meta-analysis to collect the existing PPF PK studies and provide a more accurate estimate of CYP2D6 metabolizer phenotype-dependent effects on the PPF PK. We aimed to provide findings that clinical practitioners can consult to adjust the dosage regimen to achieve optimal therapy outcomes while avoiding adverse effects of overdosing. PPF is used as a racemic mixture of S- and R- enantiomer forms. Studies have found that there are significant difference in the PK profiles of the S- and R-forms of PPF [17,18,19]. For example, the area under the plasma concentration–time curves for S-PPF were significantly higher (S/R ratio, 1.50 ± 0.17), and the apparent oral clearance was significantly lower (S/R ratio, 0.68 ± 0.07) than those of R-PPF [19]. Therefore, we also compared the PK parameters between S- and R-PPF according to CYP2D6 phenotype.

## 2. Materials and Methods

### 2.1. Data Sources and Study Selection Criteria

#### Search Strategy

This study followed the PRISMA guidelines [20]. The protocol was registered with PROSPERO (registration number CRD42021232609). We identified relevant studies through PubMed, Google Scholar, Embase, Web of Science, and Cochrane Library using the following keyword combination: “propafenone” and “CYP2D6”. The search strategy is provided in Table 1. The language restriction was English, and the literature retrieval was carried out by two independent reviewers.

The inclusion criteria were as follows: analyses of the association between the PPF PK profile and CYP2D6 phenotype or genotype in humans; reported the sample size of each phenotype; reported at least one of either the mean plus standard deviation or the median plus range or the mean plus *p*-value; and provided one or more of the following PK parameters: area under the time–concentration curve (AUC), maximum concentration (C_max_), apparent clearance (CL/F), and half-life (t_1/2_).

The exclusion criteria were insufficient information for data extraction, outcomes not in our focus of interest (e.g., studies performed in animals or that reported pharmacodynamic results only, without PK parameters, or studies in which subjects were co-administered propafenone with another drug), review articles, and other types of articles (such as case reports or expert opinions).

### 2.2. Data Extraction

The following data from each study were extracted: first author name; year of publication; PPF dosage; number of subjects and their age, sex, and status (healthy or patient); country; study duration; phenotypic/genotypic classification; and outcome data (AUC, C_max_, t_1/2_, and/or CL/F).

### 2.3. Statistical Analysis

The statistical analysis was performed using Review Manager (RevMan) [Computer program], version 5.4.1, the Cochrane Collaboration, 2020, Oxford, UK). Heterogeneity across studies was determined using the Cochrane’s Q-test and I^2^ statistic. *p*-values < 0.1 and I^2^ > 50% indicated significant heterogeneity, and a random-effects model was applied; otherwise, a fixed-effects model was applied. A *p*-value of <0.05 was considered statistically significant. Publication bias was assessed using Egger’s test where possible. The quotient between two means of pooled PK parameters of two phenotypes or two enantiomers was calculated using Filler’s method [21] with an online calculator available at https://www.graphpad.com/quickcalcs/errorProp1/ (accessed on 20 June 2022).

## 3. Results

### 3.1. Study Characteristics

**Figure 1:**
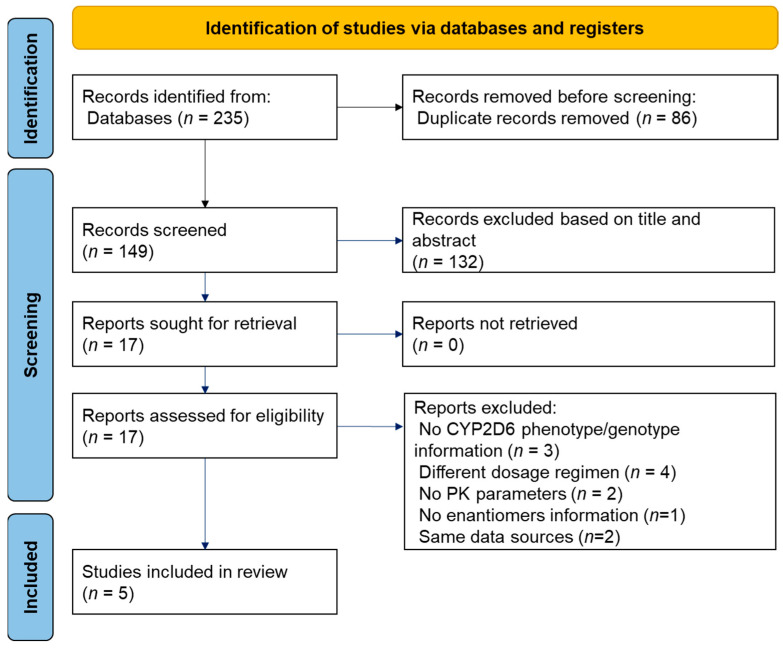
Flow chart of the selection process of all articles included in this meta-analysis.

The results of the literature screen process are presented in Figure 1. A total of 235 studies were initially identified. After an initial screen, 218 studies were excluded, owing to removing duplicates and after screening the titles and abstracts. The remaining 17 studies underwent full-text review to assess eligibility. Of these, 12 studies were excluded for the reasons presented in Table 1.

Five studies were included in the final analysis [10,16,30,31,32], with a total sample size of 56 healthy subjects.

Due to the non-linear pharmacokinetics of PPF [7], our meta-analysis was performed on the basis of the dosage regimen. Two major analyses were performed by dividing the data according to the dose: a single oral dose of 300 mg (*n* = 31) and a single oral dose of 400 mg (*n* = 25). For the 300 mg dose, the subjects were classified into two phenotypes, PMs and EMs, and a comparison was performed between these two phenotypes. For the 400 mg dose, one of the two identified studies had only an EM group; therefore, a comparison between PMs and EMs at this dose was not possible. Instead, we performed an analysis between the two PPF enantiomers (S- and R-form).

### 3.2. Meta-Analysis Results

The outcomes related to PPF PK (C_max_, AUC, CL/F, and t_1/2_) were analyzed for their association with CYP2D6 phenotype. The meta-analysis results (forest plots and related results) are provided in Table 2 and in Figures S1 and S2.

A meta-analysis of the pooled racemic data demonstrated a clear effect in all queried PK parameters. All comparisons showed significant differences (all *p* < 0.001).

#### 3.2.1. Comparison of PMs and EMs at the 300 mg Dose

At a dose of 300 mg, the PMs showed significantly higher C_max_, AUC, and t_1/2_ values compared with the EMs, with mean differences of 0.51 (95% CI: 0.25–0.76), 13.83 (95% CI: 8.48–19.19), and 9.65 (95% CI: 8.26–11.05), respectively (Table 2, Figure S1).

#### 3.2.2. Comparison of S-Form and R-Form of PPF in EMs at the 400 mg Dose

Compared with the R-form, the S-form had considerably higher C_max_ and AUC values (mean difference, 0.13 (95% CI: 0.06–0.20) and 0.61 (95% CI: 0.23–0.99), respectively) and a significantly lower CL/F (mean difference, −35.00 (95% CI: −56.63, −13.46)) (Table 1, Figure S2).

#### 3.2.3. Comparisons of the Quotient between CYP2D6 Phenotype and Enantiomers According to Dose

Table 3 and Table 4 show the mean and quotient of the PPF PK parameters according to CYP2D6 phenotype (Table 3) and PPF racemic form (Table 4).

At 300 mg, the C_max_, AUC, and t_1/2_ values were 1.10 (0.796–1.40) µg/mL, 15.9 (12.5–19.2) µg·h/mL, and 12.8 (11.3–14.3) h in PMs, respectively (Table 3). These values were 11.2-, 2.4-, and 4.7-fold higher than those in EMs.

At 400 mg (Table 4), the AUC and C_max_ values were 2.22 (1.93–2.51) µg·h/mL and 0.42 (0.365–0.475) µg/mL, respectively, for the S-form and were both approximately 1.4-fold higher than those of the R-form. The CL/F of the R-form was 127 (101–153) L/h and was 1.4-fold higher than that of the S-form.

### 3.3. Risk of Bias across Studies

A funnel plot test for asymmetry should be used only when there are at least 10 studies in a meta-analysis. When there are fewer studies, the power of the test is too low to distinguish chance from true asymmetry [33]. Thus, a funnel plot test was not performed in this meta-analysis.

## 4. Discussion

Propafenone is mainly metabolized by CYP2D6 and CYP3A4, and CYP2D6 is a well-known enzyme with polymorphisms. The CYP2D6 polymorphism leads to differences in the PK profile of PPF among phenotypes. Hence, we investigated differences in the PK parameters of PPF on the basis of the CYP2D6 phenotype.

This meta-analysis provides additional evidence of the effects of different CYP2D6 metabolizer phenotypes on the PPF PK. According to our meta-analysis, PK parameters of PPF were significantly different between PMs and EMs and between the S- and R-enantiomers (all *p*-values < 0.001). The t_1/2_ of PPF in EMs at a dose of 300 mg was 4.7-fold lower than that in PMs, which could explain the change differences in the AUC and C_max_ values between the groups, with 11.2-fold and 2.4-fold increases differences in PMs, respectively. John T. Lee et al. [27] also reported that at a dose of 300 mg, the peak concentration at steady-state in PMs was approximately 2.2-fold higher than that in EMs. The changes in PPF exposure due to CYP2D6 polymorphism can result in an underdose or overdose in patients. This can lead to a lack of treatment effect (due to underdose) or adverse effects (due to overdose) such as conduction disorders in myocardium patients (e.g., PQ prolongation, ventricular tachycardia). There are case reports of PPF toxicity, such as symptomatic bradycardia, coma, and seizures, and it had been assumed that these patients were PMs [34,35]. Therefore, the CYP2D6 phenotype should be determined before prescribing the PPF dose to ensure efficacy and avoid adverse effects.

We also found that at 400 mg, the S-form AUC was 1.4-fold higher than that of the R-form, which could be attributed to the 0.7-fold lower CL/F of the S-form compared to the R-form. Several studies have reported that PPF administered as either the S- or R-enantiomer results in the S-form CL/F that was higher than that of the R-form. If subjects were treated with PPF racemate, then the R-form CL/F was higher than that of the S-form [17,22]. This was attributed to an interaction between S- and R-enantiomers in which the R-form inhibited the elimination of the S-form [36].

Currently, the dose of many drugs is adjusted on the basis of the CYP2D6 phenotype. The average dose recommendation (D_AV_) provided by the manufacturer is based on a mixture of genetic populations. For example, the Caucasian population contains approximately 10% PMs, 40% IMs, and 50% EMs; thus,

D_AV_ = (0.1**·**D_PM_ + 0.4**·**D_IM_ + 0.5**·**D_EM_) (1)

where D_PM_, D_IM_, and D_EM_ represent the optimal doses for PMs, IMs, and EMs, respectively, and those doses were calculated on the basis of a ratio of their corresponding PK parameters (such as the AUC and CL/F) [2]. For drugs showing saturation kinetics, dose adjustments are only applied in the dose range given in the studies. PPF was reported to have non-linear kinetics, possibly due to saturation of first-pass hepatic metabolism at high dose [37,38]. Therefore, if dose adjustment is applied, it should be performed at each dose level. At present, PPF is available as a racemate. Hence, dose adjustment should be provided for the racemic mixture. However, in our analysis, at a dose of 400 mg, the PK parameters of PPF were reported for each enantiomer, and we could not combine the data from the S- and R-forms. Therefore, it is difficult to adjust the PPF dose at 400 mg on the basis of the CYP2D6 phenotype. At 300 mg, the included studies in our analysis were classified into two phenotypes only (PMs and EMs). If EMs could be further divided into two small subgroups, then it would be possible to adjust the dose. (Note that due to differences in the phenotype naming system, EMs in our analysis can cover both IMs and EMs in Equation (1).)

Our study has some limitations. First, our meta-analysis included relatively small studies that were performed in healthy participants. Therefore, caution is needed when interpreting results in the context of patients. Second, the lack of diversity of ethnic groups in this analysis could limit the generalizability of the results. Third, PPF is also metabolized by CYP3A4, but the included studies did not provide information about the metabolism carried out by CYP3A4 or the effects of CYP3A4 polymorphisms on the PPF PK parameters. Fourth, this meta-analysis covered only the effect of CYP2D6 polymorphism on the PK of PPF and did not consider pharmacodynamics. Further studies could be performed to investigate how changes in the PK parameters according to CYP2D6 phenotype affect the pharmacodynamics of PPF.

## 5. Conclusions

The results of this study have potential implications for clinical practice. The study provides evidence in support of CYP2D6 phenotyping by demonstrating a clear correlation between metabolizer phenotype and the PPF PK profile. Phenotyping patients before initiating PPF therapy would potentially avoid adverse effects or a lack of therapeutic effect. Appropriately adjusting the initial PPF dose or using of alternative drugs that are not major substrates for CYP2D6 should be considered.

## Figures and Tables

**Table 1:** Reasons for excluding 12 articles.

Reasons for Exclusion	References
No information on CYP2D6 phenotype/genotype was reported	[17,19,22]
Studies with dosage regimens were different from the other studies - 150 mg twice daily - 225 mg three times daily - 150 mg twice/three times or 75 mg three times daily - 450–600 mg/day (The other studies used 300 mg or 400 mg) Due to the non-linear pharmacokinetics of PPF, combining studies according to normalized dose is inappropriate; thus, the four above studies were excluded.	[23,24,25,26]
Two studies with a dosage of 150 mg three times per day but no identical PK parameters were reported: - One reported AUC and CL - One reported the steady-state concentration	[18,27]
No information on PPF enantiomers was reported (400 mg dose) There were four more studies with a 400 mg dose; however, PPF was clarified as R- and S-enantiomer in those studies. Therefore, study [28] was excluded.	[28]
Three studies had used the same data, thus two of them were excluded.	[12,29]

**Table 2:** Results of meta-analysis regarding propafenone pharmacokinetics.

Dose	Subgroup	Sample Size	Outcome	*p*-Value	Mean Difference (95% Prediction Interval)	Heterogeneity		References
						I^2^	Model	
300 mg	PM vs. EM	24	C_max_	<0.001	0.51 [0.25, 0.76]	0%	Fixed model	[10,16]
			AUC	<0.001	13.83 [8.48, 19.19]	60%	Random model	
			t_1/2_	<0.001	9.65 [8.26, 11.05]	0%	Fixed model	
400 mg	S-form and R-form in EM group	25	C_max_	<0.001	0.13 [0.06, 0.20]	0%	Fixed model	[30,31]
			AUC	<0.001	0.61 [0.23, 0.99]	0%	Fixed model	
			CL/F	<0.001	−35.00 [−56.53, −13.46]	0%	Fixed model	

C_max_, the maximum concentration of propafenone; AUC, area under the time–concentration curve; t_1/2_, half-life; CL/F, apparent clearance; PM, poor metabolizer; EM, extensive metabolizer.

**Table 3:** Pooled analysis of propafenone pharmacokinetics stratified by CYP2D6 phenotype at a dose of 300 mg.

	*n*	C_max_ (µg/mL)	AUC (µg·h/mL)	t_1/2_ (h)
PM	10	1.10 (0.796–1.40)	15.9 (12.5–19.2)	12.8 (11.3–14.3)
EM	21	0.453 (0.294–0.612)	1.41 (0.894–1.93)	2.73 (2.2–3.27)
Ratio PM/EM		2.42 (1.53–4.07)	11.23 (7.45–18.88)	4.68 (3.73–6.05)

Data represented as mean (95% confidence interval). C_max_, the maximum concentration of propafenone; AUC, area under the time–concentration curve; t_1/2_, half-life; PM, poor metabolizer; EM, extensive metabolizer.

**Table 4:** Pooled analysis of propafenone pharmacokinetics stratified by PPF enantiomers at a dose of 400 mg.

	*n*	C_max_ (µg/mL)	AUC (µg·h/mL)	CL/F (L/h)
S-form	25	0.42 (0.365–0.475)	2.22 (1.93–2.51)	88.4 (75–102)
R-form	25	0.30 (0.257–0.343)	1.62 (1.39–1.85)	127 (101–153)
Ratio S-/R-form		1.40 (1.15–1.72)	1.37 (1.22–1.68)	0.70 (0.540–0.917)

Data represented as mean (95% confidence interval). C_max_, the maximum concentration of propafenone; AUC, area under the time–concentration curve; CL/F, apparent clearance; S-form, R-form: two PPF enantiomers.

## Data Availability

Not applicable.

## Supplementary Materials

The following supporting information can be downloaded at https://www.mdpi.com/article/10.3390/pharmaceutics14071446/s1, Figure S1: Forest plots for association between CYP2D6 phenotypes and pharmacokinetic parameters of propafenone (A—AUC, B—C_max_, C—t_1/2_) at a dose of 300 mg. Figure S2: Forest plots for association between propafenone enantiomers and its pharmacokinetic parameters (A—AUC, B—C_max_, C—CL/F) at a dose of 400 mg.
