# Supplementary material for: The Effect of CYP2D6 Phenotypes on the Pharmacokinetics of Propafenone: A Systematic Review and Meta-Analysis
Source: Pharmaceutics. 2022 Jul 11;14(7):1446. doi: 10.3390/pharmaceutics14071446 (PMC9324789; doi:10.3390/pharmaceutics14071446)
Supplement: Supplementary file 1 [file pharmaceutics-14-01446-s001.zip › pharmaceutics-1743984-supplementary.pdf]

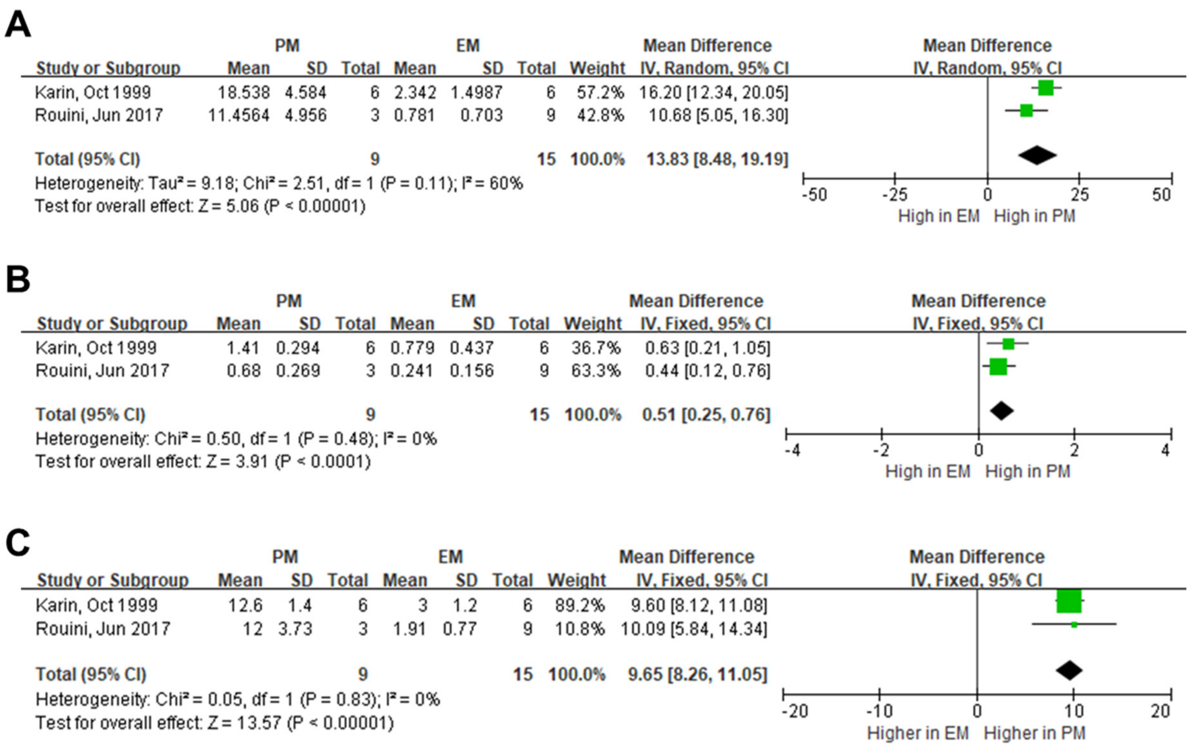

Figure S1. Forest plots for association between CYP2D6 phenotypes and pharmacokinetic parameters of propafenone (A - AUC, B - C_{max}, C - $t_{1/2}$) at dose 300 mg.

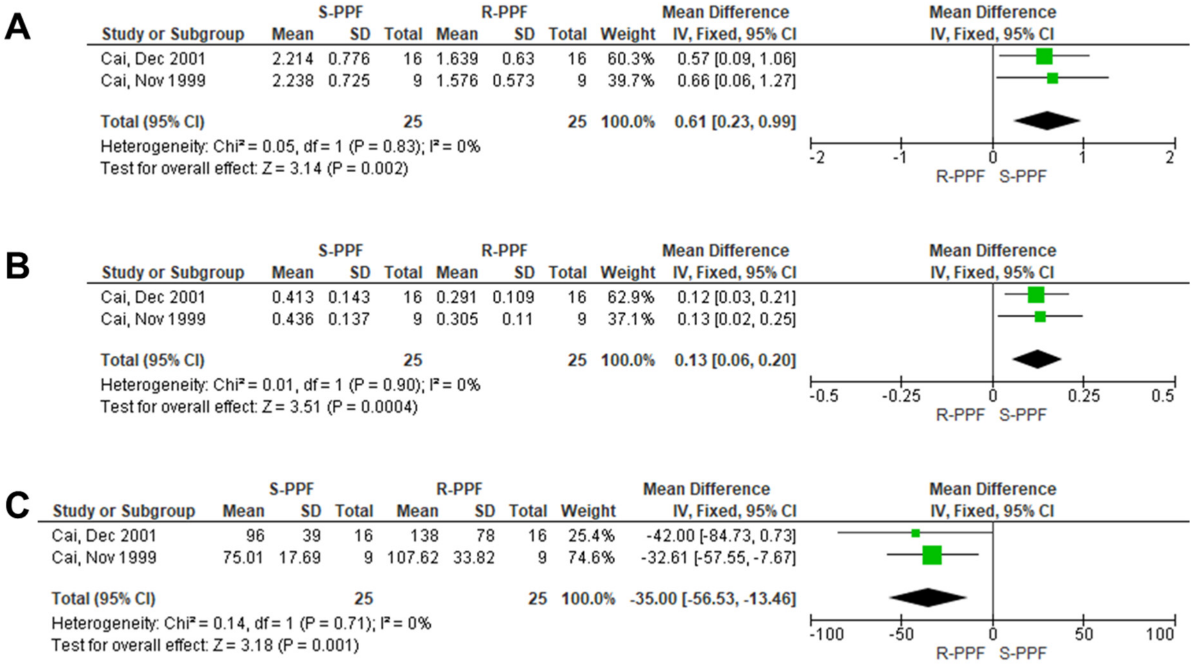

Figure S2. Forest plots for association between propafenone enantiomers and its pharmacokinetic parameters (A - AUC, B - C_{max}, C – CL/F) at dose 400 mg.
